# Effect of leadership styles on turnover intention among staff nurses in private hospitals: the moderating effect of perceived organizational support

**DOI:** 10.1186/s12913-024-10674-0

**Published:** 2024-02-14

**Authors:** Surabhila Pattali, Jayendira P. Sankar, Haitham Al Qahtani, Nidhi Menon, Shabana Faizal

**Affiliations:** 1 College of Administrative and Financial Sciences, University of Technology Bahrain, Salmabad, Kingdom of Bahrain; 2 Vice President of Academic Affairs, University of Technology Bahrain, Salmabad, Kingdom of Bahrain

**Keywords:** Transformational leadership, Authentic leadership, Perceived organizational support, Turnover intention

## Abstract

Leadership styles have often been proven to support employees in performing their duties better and with more efficiency while enabling them to have extended organizational tenures. Staff nurses are an essential resource of hospitals to ensure proper administration and quality patient health care. The study aims to determine how transformational and authentic leadership styles affect the staff nurses’ turnover intention in private hospitals. In addition, it also finds the moderating effect of perceived organizational support. An explanatory quantitative research design with a cross-sectional investigation and a stratified sampling strategy was used for the study. Data from 296 nurses from the eight chosen private hospitals in the Kingdom of Bahrain were gathered using a questionnaire with 24 items. Smart-PLS was employed to conduct PLS-SEM (partial least squares structural equation modeling) to measure direct and indirect effects. The result indicates that transformational, authentic leadership styles and perceived organizational support significantly negatively affect nurses’ turnover intention. The study confirms the negative moderating effect of perceived organizational support between transformational leadership and turnover intention and the positive moderating effect of perceived organizational support between authentic leadership and turnover intention. Managers should concentrate on the leadership style to avoid its impact on turnover intention. By considering human resource practices such as communication and training strategies to cope with the negative effect of turnover intention, organizations can enhance employee engagement, improve job satisfaction, and foster a more stable and productive work environment. The present research revealed the adverse impact of turnover intention within hospitals by examining its association with leadership styles. The research made a significant contribution to the existing literature by delving into the impact of leadership styles on turnover intention, focusing on the moderating effect of perceived organizational support. The study’s findings shed light on the intricate relationship between leadership practices and employee turnover, providing valuable insights for both scholars and practitioners in the field. The study used a cross-sectional design to collect data and ensured the absence of standard method variance. The research enhanced the social dominance theory (SDT) by examining how perceived organizational support moderates the relationship between leadership styles and turnover intention.

**Supplementary Information:**

The online version contains supplementary material available at 10.1186/s12913-024-10674-0.

## Introduction

Leadership is an act of directing the employees in an organization. Usually, leaders ought to encourage employees and allocate duties to personnel or groups toward overall goal attainment. Their style characterizes a leader's behavior when addressing organizational difficulties [1]. Distinct leaders have distinct leadership styles. Each leadership style contains favorable and unfavorable traits, according to Garrison (2022) [2]. The manager employs different leadership styles to bring workplace changes [3]. To withstand in the globally competitive world, leaders experiment with varying types of leadership to achieve organizational goals and motivate employees [4]. Transformational leaders (TLE) insist that their followers give their most capacity by setting clear directions based on their perceptions [5]. Leadership in hospitals is aligning the employees with the leaders' view in achieving organizational goals [6]. In hospitals, nurses are directly involved in the patient's care, so there is a need to create a manager-friendly environment to enhance the staff nurses' commitment [7]. Nurse managers are responsible for retaining nurses. In this case, leadership style determines whether the nurses stay in the same hospital, request transfer to other units, or seek jobs elsewhere [8].

Authentic leaders (ALE) express behavioral attitudes and encourage followers to demonstrate a positive mindset in the workplace. These leaders can create an enjoyable work environment that enhances management transparency, information disclosure, internalizing moral ideals, and SAW [9]. When an organization values employees' work, workers' well-being and socio-emotional needs are met [10]. Perceived organizational support (POS) will make employees feel the organization considers their values [11]. When analyzing employee turnover, turnover intention (TIN) can be used to gauge an employee's decision-making process regarding whether or not to quit the company during the specified time. Identifying the TIN is most important for an organization to substantiate the employee leaving the company, according to Puhakka et al. (2021) [12]. TIN is not limited to any particular organization [13]. Experts believe TIN is meant for organizations with less flexible work environments [14]. The negative side of employee turnover is the organization's human and financial costs. Human resource costs incurred due to employee turnover include hiring, new employee selection, orientation, and so on [15].

In healthcare organizations, employees (administrators, doctors, and nurses) play a vital role in ensuring the social system. Recently, the scarcity of healthcare workers has become a significant concern. As per WHO (2016) [16], the Commission on Health Employment and Economic Growth's study report mitigates the attrition of 18 million health workers. According to WHO, to achieve sustainable development goal 3, considering the entire global healthcare staff, there will be a demand for 9 million more nurses and midwives by 2030, out of which 50% are nurses [17]. This signifies that there will be a substantial nurse shortage in the healthcare business, which will continue to be costly for the industry until new generations of nurses enter the market or present nurses are given appropriate incentives to stay in the sector. The study mainly focuses on the healthcare sector, the staff nurses in the Kingdom of Bahrain, a Western Asian island nation with a population of 17,83,983 people and a 2.75 per cent annual population growth rate [18]. As per the report [19], the GDP of Bahrain is higher than the global GDP average. As per O’Neill (2022) [20], in 2019, Bahrain’s per capita income was $44,330. Further, according to NHRA (2022) [21] report, Bahrain has 10,030 nurses registered with National Health Authority (NHRA).

There is a need for a firm retention policy in the healthcare sector to strengthen its community services, increase revenues, ensure quality patient care and productivity, and preserve hospital operations [22]. Moreover, increasing nurse turnover will imply human resource costs and compromise treatment quality [23]. Thus, the study examines nurses’ TIN through the influence of leadership styles. The primary aim of this paper is to determine the relationship between leadership style and staff nurses' intentions to leave private hospitals and identify the moderating role of perceived organizational support. The main subjects of this study are the influential role of leadership styles and nurses’ TIN. The study considered two leadership styles: ALE and TLE. The framework of the study is derived based on practical experience, various research discussions, and published research. The extensive literature on the TIN of hospital employees is available, but studies specific to private hospitals in Bahrain are rare. Thus, to fill the research gap, the research focused on the influence of leadership styles on the TIN in private hospitals in Bahrain with the moderating effect of perceived organizational support.

Social dominance theory (SDT) [24] discussed group-based dominance and was utilized to measure the leadership style on turnover intention through perceived organizational support. The key objective of the current study is to examine the relationship between TLE (individual consideration (ICO), idealized influence (IIN), inspirational motivation (IMO), and intellectual stimulation (ISI)) [25] and ALE (balanced processing (BPR), internalized moral perspective (IMP), relational transparency (RTR), and self-awareness (SAW)) [26, 27], which in turn influences staff nurses' intention to leave private hospitals and the moderating effect of perceived organizational support. Moreover, this study contributes theoretically by delving into the intricate dynamics between leadership styles, particularly TLE and ALE, and staff nurses' TIN in the context of private hospitals in Bahrain. Recognizing the scarcity of research in this specific domain, the research addresses a crucial gap by examining the influence of leadership styles on nurses' TIN, considering Bahrain's unique socio-economic and healthcare landscape. Moreover, the study introduces the concept of POS as a moderating factor in the relationship between leadership styles and TIN. Grounded in SDT, the research explores how TLE and ALE, with their distinct dimensions, impact nurses' intentions to leave and how POS mitigates this effect. By doing so, the paper not only enriches the literature on turnover intentions but also provides valuable insights for healthcare organizations in Bahrain aiming to enhance staff retention strategies amid the global shortage of healthcare workers.

## Literature review

Two leadership philosophies- TLE and ALE- have been studied to determine their effects on TIN among nurses. The intention to leave the job has significantly lessened because of the leadership methods [28]. On the contrary, laissez-faire leadership increases the intention of nurses to leave the job [29]. Leadership style is closely connected to work value and indirectly influences employees' TIN. Direct or indirect leadership behaviors affect organizational commitment [30]. Management support will enhance social connections and employee loyalty in the workplace. Kohll (2018) [31] represented that a pleasant work environment will increase efficiency and productivity and may reduce employees' TIN.

Major workplace problems like low job satisfaction, poor nurse-physician leadership, stressful working environment, and complex workload will lead nurses to leave the hospital [29]. Nurses' TIN is not based on culture, wealth, and household factors; Majeed & Jamshed (2021) [32] mentioned that we can't ignore the view of nurses’ TIN due to the leadership style, which varies from country to country. According to Doherty & Hunter Revell (2020) [33], there are few studies on nurses dealing with leadership styles (ALE). Therefore, intense research on leadership style is necessary to ensure the retention of skilled employees in the healthcare system [34].

In leadership, two significant styles, TLE and transactional leadership, are two familiar styles that leaders usually exhibit. Much literature is available on organizational behavior and TLE [35]. According to Piccolo & Colquitt (2006) [36], some studies insist on turnover over the intention to innovation, work satisfaction, leader effectiveness, objective and subjective performance ratings, and quality improvements. Outcomes of transactional leadership will influence perceptions of leadership trust and fairness. The situation is the deciding factor in leadership styles [37].

The association between leadership behavior and employee TIN was examined by [38]. According to the research, there is a negative correlation between turnover choice and a favorable influence on the intention to leave. Tian et al. (2020) [39] revealed little effect on turnover and transactional leadership. In an organization, TLE will enhance the followers' potential to achieve significant results [40]. Ethical leaders enable the highest possible delivery and encourage engagement in behaviors through societal standards and increasing attitudes [41].

Over the past ten years, ALE has become a new administration component. ALE must stay abreast of recent advancements to help employees feel connected and find meaning in their work [42]. Usually, ALE is attached to organizational values to build credibility; followers trust and respect the leaders’ credibility [43]. Likewise, ALE encourages followers to speak up and form equal-opportunity relationships. Further, Duarte et al. (2021) [44] stressed that ALE is crucial for enhancing organizational effectiveness in perceived organizational performance and job satisfaction, reducing TIN and negativity against top management.

Table 1 represents the constructs, variables, and source summaries of the previous studies according to the key variables in this study.

**Table 1:** Summary table of research variables

Construct	Variables	Sources
Transformational Leadership (TLE)	Individual Consideration (ICO)	(Bass & Avolio B. J., 1994)
	Idealized Influence (IIN)	
	Inspirational Motivation (IMO)	
	Intellectual Stimulation (ISI)	
Authentic Leadership (ALE)	Balanced Processing (BPR),	(Avolio & Gardner, 2005; Kernis, 2003)
	Internalized Moral Perspective (IMP),	
	Relational Transparency (RTR)	
	Self-awareness (SAW)	
Perceived Organizational Support (POS)		(Eisenberger et al., 1986)

## Theoretical background and hypotheses development

### Social dominance theory

Social dominance theory (SDT) [24] discussed group-based dominance and was utilized to measure the leadership style on turnover intention through perceived organizational support. According to Nicol et al. (2011) [45], perceived organizational support moderates the relationship between leadership style and turnover intention, commitment, and satisfaction. Social dominance orientation deals with the employees leaving the organization based on their own beliefs of the organization with perceived organizational support [46].

### Transformational leadership and turnover intention

The four elements of the TLE style are ICO, IIN, IMO, and ISI. Khalil & Sahibzadah (2017) [47] state that ICO and overall satisfaction will strongly influence job satisfaction. The critical components under ICO are recognizing the employee's contribution, mentoring, fulfilling individual employee needs, and open communication. IIN is decided based on the charisma of the leader. For the benefit of others, leaders make personal sacrifices and override their SAW. IIN adversely correlates with employee TIN, implying that IIN will lessen employee TIN [36].

Inspirational leadership refers to the leaders' ability to sense purpose and motivation and inspire confidence in the followers to achieve goals and overcome challenges [45]. In inspirational leadership, the leader expresses the task at hand, values self, focuses on others, is honest, has high integrity, portrays steady leadership and excellent communication, and displays solid IMO [48]. Further, inspirational leaders encourage employees' imagination, ability, innovation, creativity, critical thinking, and problem-solving rationally [49]. An increase in overall employee satisfaction in an organization will reduce TIN. Kim & Kim (2018) [50] stressed that ISI positively influences job satisfaction. Accordingly, it is hypothesized that:

#### H1. There is a significant negative effect of TLE on nurses' TIN in Bahrain’s private hospitals

### Authentic leadership and turnover intention

In ALE, leaders deal with followers with transparency in information sharing and ethical behavior by accepting the follower's ideas in decision-making [51]. BPR deals with the qualities of managers by increasing organizational commitment, which will reduce TIN [52]. Further, by including employees in decision-making, BPR avoids distortion, objective analysis, ignoring information, and exaggeration [53]. Leaders maintain consistency in IMP to demonstrate justice, solid professional practice, core principles, and judgments [54]. Managers’ quality will increase organizational commitment through IMP and reduce TIN [55].

RTR includes critical components that leaders display, such as developing confidence, sharing genuine trust, sharing feelings, and appropriate emotions [56]. Malekzadeh F (2019) [57] mentioned that RTR hurts TIN and job insecurity. Thus, an increase in RTR will reduce the TIN. In leadership, SAW will prevent the leader from comparing the characteristics of one with another; all the employees are treated uniquely by the leaders [44]. Moreover, the SAW of ALE will improve social functioning and emotion. According to Giao et al. (2020) [58], SAW increases organizational commitment and reduces TIN. Therefore, it is hypothesized that:

#### H2. There is a significant negative effect of ALE on the nurses’ TIN in Bahrain’s private hospitals

### Perceived organizational support and turnover intention

Employees' perception of the organization's commitment to their socio-emotional needs, fulfilment, and well-being is the idea of perceived organizational support (Meyers et al., [59]). Moreover, perceived organizational support will increase job satisfaction, leadership, working conditions, and organizational performance and reduce absenteeism [60]. Perceived organizational support considerably influences employee satisfaction and work engagement and negatively impacts TIN [61]. According to Srivastava & Agrawal (2020) [62], perceived organizational support is closely connected to leadership and, as a moderator, reduces TIN. Accordingly, it is hypothesized that:

#### H3. There is a significant negative effect of Perceived organization support on nurses' TIN among Bahrain’s private hospitals

### The moderating role of perceived organizational support

Nurses may face time constraints and a high workload due to a shortage of staff caused by increased nurse TIN [63]. To overcome employee turnover constraints, nursing managers must practice ALE and TLE to improve the work environment and support staff nurses [64]. Leadership styles will help the staff nurses and managers avoid employee conflict, rectify the unhealthy hospital environment, open mutual communication and trust, and reduce the nurses' intention to leave the current hospital [65]. Therefore, it is hypothesized that:

#### H4. There is a significant moderating effect of perceived organizational support between TLE and nurses' TIN in Bahrain’s private hospitals

##### H5. There is a significant moderating effect of perceived organizational support between ALE and nurses' TIN in Bahrain’s private hospitals

Overall, the theoretical contributions pertain to examining various leadership styles and their impact on turnover intention among nurses in Bahrain's private hospitals. Further, the theoretical contributions encompass the application and integration of SDT, TLE, ALE, and the role of POS in understanding and predicting turnover intention among nurses, focusing on Bahrain’s private hospitals. The study also introduces hypotheses that can be empirically tested to validate these theoretical propositions.

**Fig. 1:**
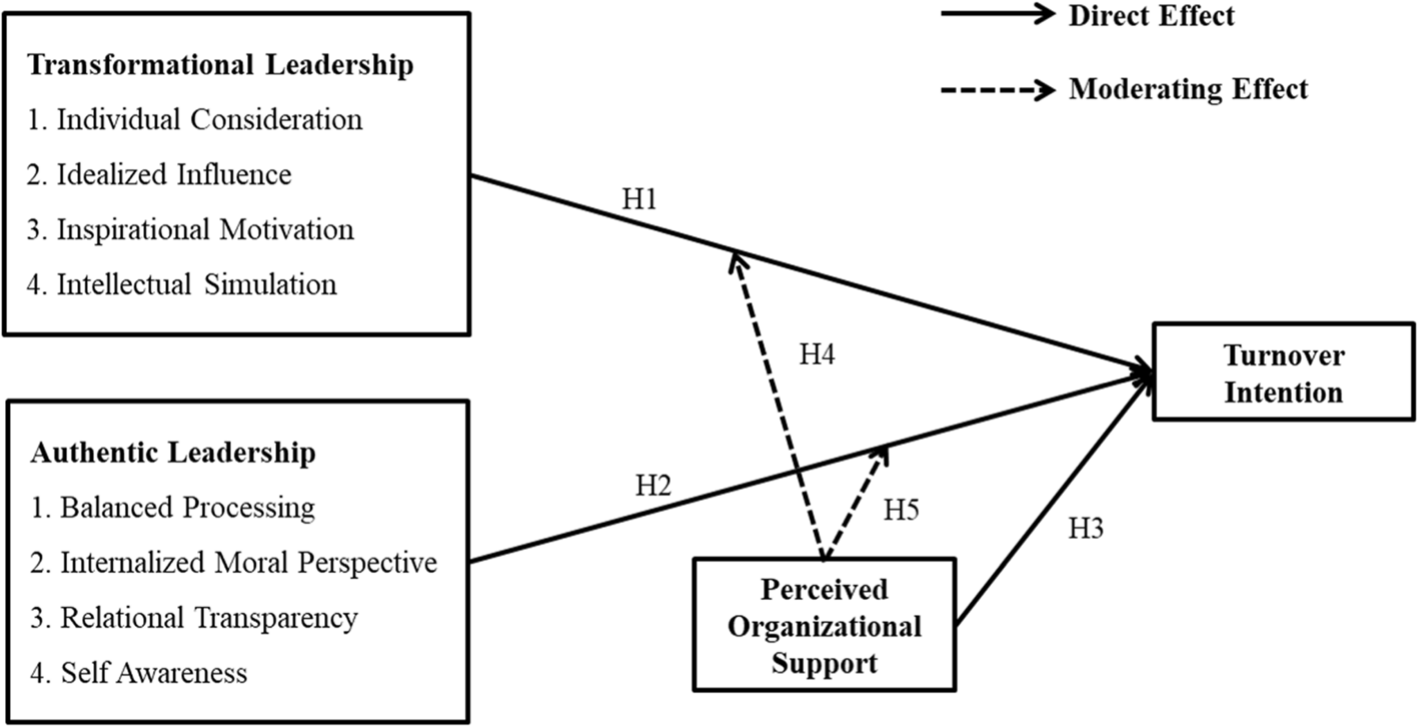
Conceptual framework

Figure 1 represents:

H1—the significant negative effect of TLE (ICO, IIN, IMO, and ISI) on nurses' TIN.

H2—the significant negative effect of ALE (BPR, IMP, RTR, and SAW) on nurses' TIN.

H3—the significant negative effect of perceived organizational support on nurses' TIN.

H4 and H5—moderating effect of perceived organizational support between TLE and ALE on nurses' TIN.

## Materials and methods

### Area of study

The Kingdom of Bahrain, commonly known as Bahrain, is an island nation in Western Asia. Bahrain consists of 83% of the nation’s landmass, a small archipelago of 50 natural islands, and an additional 33 artificial islands. Between Qatar and Saudi Arabia's northeastern shore, which the King Fahd Causeway connects, is where Bahrain is located. The population of Bahrain is 1,783,983 people, with a 2.75 percent annual population growth rate [18]. Further, Bahrain is the third-smallest country in Asia after Maldives and Singapore, with 290 square miles of area. The capital of Bahrain is Manama. As per NHRA (2022) [21], there are 20 private hospitals in Bahrain. Of these, 15 hospitals are in the capital governorate, 1 in Muharraq, 1 in the Northern governorate, and 3 in the Southern governorate. The study utilized a cross-sectional investigation with a stratified sampling strategy. Eight private hospitals in Bahrain were chosen for the study: 3 from the central, 1 from Muharraq, 1 from the Northern governorate, and 3 from the Southern governorate based on the number of bed facilities available in the hospitals.

### Sampling and data collection

The total population of the study was based on the number of registered nurses working in private hospitals in Bahrain. A total of 1355 registered nurses work in 20 private hospitals in Bahrain [21], of which 813 were in the selected eight private hospitals. For this study, the sample included 300 respondents. Expecting a 10% error (30 respondents) in the response, 330 questionnaires were distributed. The completed responses of 296 respondents were considered for analysis. As per Krejcie & Morgan (1970) [66], the sample required for perfect analysis is between 260–265, but the study collected more than the required number of samples. A random sampling procedure was used to determine respondents for data collection. An index number has been created for the nurses in the eight selected hospitals to ensure personal information protection and confidentiality. The Excel spreadsheet produced an index number with the random list, and the respondents were requested to complete the printed questionnaire manually. Nationalities classified into Bahrainis and non-Bahrainis (Arabs and Non-Arabs) Therefore, some questions were translated into the Arabic with the help of the human resource manager and the data collector.

### Measurement and method

The questionnaire was adopted from the different authors based on the variables (Appendix). Transformational leadership style questions were adopted from the study by Alban-Metcalfe & Alimo-Metcalfe (2000) [67]. Authentic leadership questions were adopted from the study by Cervo et al. (2016) [68]. Perceived organizational support questions were adopted from the study by Kurtessis et al. (2017) [9]. Turnover intention questions were adopted from the study by Bothma & Roodt (2013) [69]. Further, the nurses are asked to rate the extent to which their immediate superior is TLE or ALE. The questionnaire has been divided into 4 parts. TLE (ICO, IIN, IMO, and ISI) with four questions, ALE (BPR, IMP, RTR, and SAW) with four questions, POS with eight questions, and TIN with six questions. A five-point Likert scale was used to score the responses to the questions, with “5-strongly agree, 4-agree, 3-neutral, 2-disagree, 1-strongly disagree.” MS Excel was used to arrange the data, and SPSS 25.0 was used for the initial analysis.

Since it enables them to examine large models with numerous structural paths, latent variables, indicators, and constructs without focusing on data distributional assumptions, the structural equation model using Smart-PLS is currently quite popular among researchers. Additionally, PLS-SEM (partial least squares structural equation modeling) is a causal-predictive approach to SEM that stresses prediction in estimating statistical models, whose structures are intended to provide causal explanations, according to Sarstedt et al. (2019) [70]. PLS-SEM also has higher prediction power than CB-SEM [70]. Therefore, the present study used the PLS-SEM to examine the hypotheses based on the complexity of the model employing the moderation effect. As a result, the bootstrap technique (5000) was used to explore the significance of correlations after the model's reliability. Initially, data validation was done using reliability markers, internal consistency, convergent validity, and discriminant validity.

## Results

### Data normality

In testing the data normality, Hair et al. (2010) [71] and Kline (2015) [72] recommend testing the Skewness and Kurtosis of the data.

Table 2 shows that the Skewness values are (-0.083 to 0.043), and the Kurtosis values are (-1.219 to -0.935), indicating data normality. As per the recommended values of Byrne (2016) [73], the Skewness values are between -2 to + 2, and Kurtosis values are between -7 to + 7 for standard data.

**Table 2:** Descriptive statistics and data normality

*Variables*	*Min*	*Max*	*Skewness*	*Kurtosis*
ALE	-1.975	1.378	-0.083	-1.052
POS	-2.141	1.438	0.043	-0.935
TIN	-1.828	1.380	-0.012	-1.192
TLE	-1.914	1.334	-0.061	-1.219

### Goodness of model fit

Before analyzing using the structural equation model, carrying out the Goodness of model fit is mandatory to assess the data fit and inference statistics. An approximate model fit needs to be utilized to evaluate the model fit [74, 75].

Table 3 reveals that the standardized root means square residual (SRMR) is 0.078; as suggested by Dijkstra & Henseler (2015) [75], the calculated SRMR value should be less than 0.1 to be a good fit. Also, d_ULS and d_G are 1.559 and 0.998. As per Hair et al. (2017) [76], the calculated d_ULS and d_G values should be < 95% to be a good fit. All the set criteria are being met, so the model attained a good fit.

**Table 3:** Goodness of model fit

Model fit	Values
SRMR	0.078
d_ULS	1.559
d_G	0.998
Chi-square	1437.313
NFI	0.687

### Reliabilities and validities

Smart-PLS 4 was utilized to check the validity and reliability of the collected data using the confirmatory factor analysis (CFA).

Hair et al. (2017) [76] state that composite reliability (CR) should be higher than 0.70, and the average variance extracted (AVE) should be higher than 0.50 to ensure the validity and reliability of the collected data. Fornell & Larcker (1981) [77] state that the degree of shared variance between latent variance should be less than 0.90. Table 4 revealed that all the composite reliability (CR) values are higher than 0.50. The average variance extracted (AVE) values are higher than 0.50, and Fornell & Larcker values are less than 0.90, proving that the collected data are reliable and valid.

**Table 4:** Construct reliability, validity and fornell-larcker test of discriminant validity

	**CR**	**AVE**	**ALE**	**POS**	**TIN**	**TLE**
ALE	0.861	0.608	**0.780**			
POS	0.896	0.520	0.821	**0.721**		
TIN	0.910	0.629	0.772	0.805	**0.793**	
TLE	0.878	0.644	0.776	0.773	0.718	**0.802**

Henseler et al. (2015) [78] state that, to assess the discriminant validity, it is mandatory to test the Heterotrait-Monotrait Ratio of Correlations (HTMT), and the values should be less than 0.90. Table 5 Heterotrait-Monotrait Ratio of Correlations (HTMT) values proved discriminant validity.

**Table 5:** HTMT results

	**ALE**	**POS**	**TIN**	**TLE**	**POSxTLE**	**POSxALE**
ALE						
POS	0.882					
TIN	0.834	0.815				
TLE	0.871	0.810	0.842			
POS x TLE	0.125	0.121	0.109	0.077		
POS x ALE	0.112	0.151	0.079	0.078	0.885	

### Structural equation modelling (SEM)

In ensuring structural validity, SmartPLS software was performed for PLS-SEM confirmatory factor analysis (CFA). Using PLS-SEM, Hair et al. (2019) [79] represented extracted four-factor (EFA) to link latent constructs with observation indicators. Further, confirmatory analysis for non-normal distribution is suitable for indicator and endogenous and exogenous variables [70].

Hair et al. (2019) [79] suggested that the *p*-value must be less than 0.5. [70] The variance inflation factor should be less than 4 using multi-collinearity calculation. Table 6 represents the factor loading, and the values of p are less than 0.5, and VIF values are less than 4, reflecting the model is a good fit and proceeding with the path coefficient analysis.

**Table 6:** Factor loading

	**Factor loading**	**T statistics (|O/STDEV|)**	***P* values**	**Collinearity statistics (outer-VIF)**
BPR	0.837	49.106	0.000	1.781
ICO	0.806	39.456	0.000	1.735
IIN	0.835	38.865	0.000	2.081
IMO	0.790	32.848	0.000	1.743
IMP	0.723	24.404	0.000	1.409
ISI	0.777	30.555	0.000	1.535
POS1	0.783	32.849	0.000	2.275
POS2	0.690	19.572	0.000	1.706
POS3	0.558	10.102	0.000	1.460
POS4	0.685	21.823	0.000	1.562
POS5	0.721	24.705	0.000	1.847
POS6	0.756	28.076	0.000	2.017
POS7	0.761	30.912	0.000	2.289
POS8	0.788	35.294	0.000	2.169
RTR	0.802	33.548	0.000	1.738
SAW	0.753	29.903	0.000	1.565
TIN1	0.813	38.872	0.000	2.375
TIN2	0.823	37.979	0.000	2.271
TIN3	0.830	46.963	0.000	2.240
TIN4	0.724	19.951	0.000	1.647
TIN5	0.818	39.239	0.000	2.173
TIN6	0.744	26.588	0.000	1.785

**Fig. 2:**
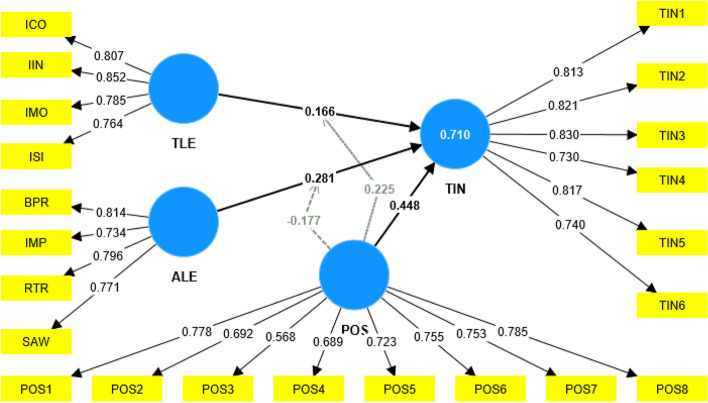
Path values

Figure 2 represents the path value. The R^2^ value for the estimated equation is 0.710. The current research model describes 71.0% of the TIN.

**Fig. 3:**
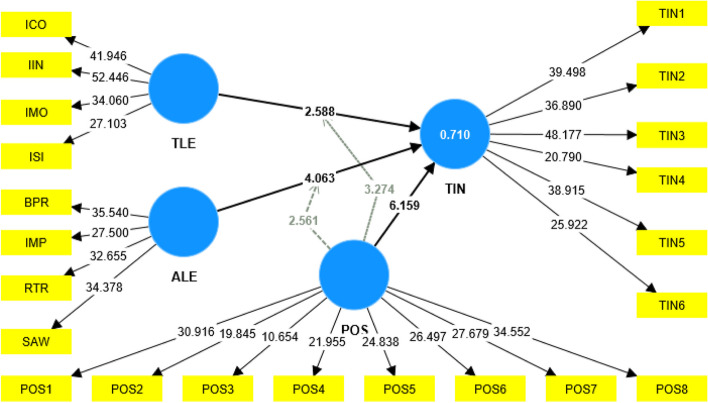
Bootstrapping (t-values)

Figure 3 represents the bootstrapping t-value results of PLS bootstrapping of the structural relationship.

In Table 7, path coefficient values indicate that H1 is supported (β = 0.166, *p* < 0.05), and the t value of 2.588 is above the 98.0% (2.327 at ∞ degrees of freedom) confidence level. Hypothesis H1 is supported, confirming that TLE has a significant negative effect on nurses' TIN, which means an increased and efficient TLE will reduce the nurses' TIN. H2 is supported (β = 0.281, *p* < 0.01), and the t value of 4.063 is above the 99.9% (3.291 at ∞ degrees of freedom) confidence level. Hypothesis H2 is supported, confirming that ALE has a significant negative effect on nurses' TIN, which means an increased and efficient ALE will reduce the nurses' TIN. H3 is supported (β = 0.448, *p* < 0.01), and the t value of 6.159 is above the 99.9% (3.291 at ∞ degrees of freedom) confidence level. H3 is supported, which confirms that perceived organizational support has a significant negative effect on nurses' nurses' TIN. An increased and efficient perceived organizational support will reduce the nurses' TIN.

**Table 7:** Path coefficient

	**Beta coefficient**	**T statistics**	***P* values**	**Decision**
ALE—> TIN	0.281	4.063	0.000	Supported
POS—> TIN	0.448	6.159	0.000	Supported
TLE—> TIN	0.166	2.588	0.010	Supported
POSxTLE- > TIN	0.225	3.274	0.001	Supported
POSxALE- > TIN	-0.177	2.561	0.010	Supported

H4 is supported (β = 0.225, *p* < 0.01), and the t value of 3.274 is above the 99.8% (3.091 at ∞ degrees of freedom) confidence level. Hypothesis H4 is supported, which confirms that perceived organizational support has a moderating effect between TLE and TIN. An increased and efficient perceived organizational support will help reduce the nurses' TIN through a TLE. H5 is supported (β = -0.177, *p* < 0.05), and the t value of 2.561 is above the 98.0% (2.327 at ∞ degrees of freedom) confidence level; Hypothesis H5 is supported, which confirms that perceived organizational support has a moderating effect between ALE and TIN, which means an increase and efficient perceived organizational support will help in reducing the nurses' TIN through ALE.

## Discussion

Employee turnover can negatively impact employees and organizations. Healthcare organizations must emphasize preventing high employee turnover [80]. Understanding the critical intentions for nurses’ turnover is central to addressing organizations' high turnover rate. The current business trends and the intense competition have forced organizations to frame different strategies to bring the turnover rate on the count [81]. This has paved opportunities for scholars to work on established human resource management practices and provide future research directions [82].

This study confirms that the TLE has a significant negative effect on nurses’ TIN (beta value = 0.166). Among the four components of TLE, IIN got the highest beta value of 0.852 (85.2%), ICO got a beta value of 0.807 (80.7%), IMO with a beta value of 0.785 (78.5%), and ISI got the lowest beta value of 0.764 (76.4%). IIN adversely correlates with employee TIN, implying that IIN will lessen employee TIN [36]. The support and orientation of a TLE give ICO to the employees so they can develop their strengths. This eventually results in the workers staying within the company for a long time, ultimately lowering the risk of quitting that organization shortly [44].

Furthermore, a TLE, out of IMO, considers the moral and ethical consequences of decisions. This will influence the employees and may significantly avert the undesired behavior of employees, including TIN [48]. Similarly, a TLE is intended to increase employee motivation by fostering a sense of shared ethics and a positive ethical identity, encouraging them to work for the firm for as long as feasible. Lastly, a TLE leader induces ISI by reexamining critical assumptions to question their appropriateness [81]. This study thus confirms the findings in the previous literature that claimed a significant negative effect of TLE and the nurses' TIN of employees in an organization.

Hypothesis 2 of the study confirms that the ALE has a significant negative effect on nurses’ TIN (beta value = 0.281). Among the four components of ALE, BPR got the highest beta value of 0.814 (81.4%), RTR has a beta value of 0.796 (79.6%), SAW a beta value of 0.771 (77.1%), and IMP has the lowest beta value of 0.734 (73.4%). The BPR style of an ALE, through which the leader solicits sufficient opinions and viewpoints before making essential decisions, gives added support to the employees [50]. They feel an elevation of trust, faith, and optimistic hope. Furthermore, the IMP of a leader, through which he sets an environment of high standards for moral and ethical conduct, helps induce positive attitudes and behaviors in the employees [54]. Interpersonal solid relationships built by ALE with employees also help reduce value conflicts, which buffers their negative thoughts and intentions [57]. Thus, the study concludes that ALE negatively predicts nurses’ TIN.

Hypothesis 3 of the study confirms that perceived organizational support has a significant negative effect on nurses’ TIN (beta value = 0.448). Perceived organizational support will increase job satisfaction, leadership, working conditions, and organizational performance and reduce absenteeism [60]. Hypothesis 4 of the study, confirms the negative moderating effect of perceived organizational support between TLE and TIN (beta value = 0.225). As per Matande et al. (2022) [83], perceived organizational support positively influenced TLE on TIN. Hypothesis 5 of the study confirms a positive moderating effect of perceived organizational support between ALE and TIN (beta value = -0.177). According to Baykal (2020) [84], perceived organizational support negatively influences ALE on nurses' TIN.

The extent to which the organization values employee contribution, appreciation for the extra effort by employees, and concern for the employees' well-being are all positive supporting factors that reduce their intentions to leave an organization. Further, the study confirms that perceived organizational support significantly moderates between TLE and ALE. Thus, TLE and ALE influence employees’ resilience positively, and there is an association between leadership style and the TIN of nurses.

## Implications

### Theoretical implications

The study contributes to theoretical advancements by grounding itself in SDT, shedding light on how TLE and ALE impact nurses' turnover intentions. The research identifies a theoretical gap, suggesting that previous studies may have overlooked the nuanced dimensions of these leadership styles in relation to turnover intentions. The introduction of POS as a moderating factor further enriches the theoretical framework, emphasizing the need for a more comprehensive understanding of the mechanisms at play in healthcare organizations.

### Practical implications

For healthcare organizations in Bahrain, the study provides practical insights into enhancing staff retention strategies. The practical gap identified suggests a lack of specific guidance for healthcare organizations in the region regarding effective strategies for retaining healthcare workers. By focusing on leadership styles and introducing the concept of POS, the study offers actionable recommendations to address turnover intentions, thereby aiding healthcare organizations in developing targeted and effective retention strategies.

### Managerial implications

Managers in the healthcare sector are urged to consider the findings as they frame practices and policies related to employee acquisition, development, and retention. The study underscores the role of leaders in adopting specific leadership styles, such as TLE and ALE, to reduce employee turnover. Managers can use this information to build shared values that align with both patient care needs and the business requirements of health organizations, fostering a work environment conducive to employee retention.

### Social implications

On a societal level, the study advocates for the creation of healthcare environments that prioritize respect, collegiality, and professionalism. Recognizing the direct reflection of employee quality in patient care, governments and agencies are encouraged to implement policies that enhance the overall work culture in healthcare. By addressing the social implications, the research contributes to the betterment of healthcare systems and the well-being of both healthcare professionals and patients.

### Economic implications

The economic implications of the study are significant, especially considering the financial burden of employee turnover in the healthcare sector. Employee turnover incurs various costs, and in a resource-scarce industry like healthcare, these costs take on unique significance. The study suggests that adopting effective leadership styles, informed by the research findings, can mitigate the economic impact of turnover. As employee retention is essential for organizational performance and economic stability, the research provides valuable insights for healthcare organizations and contributes to the broader economic health of the nation.

## Conclusion

Leadership creates initiatives to motivate workers and provides responsibilities for an individual or group to achieve a goal. The retention rate of an organization is a determinant of the success or failure of the firm. Healthcare management must foster an effective leadership style because recent research indicates that one of the top three reasons why workers quit their jobs has to do with an ineffective managerial approach. Therefore, this research has highlighted the styles of leadership management that can be adapted to retain the workforce. Ultimately, this research offers current information into Bahrain’s healthcare industry to help prevent nurses’ TIN, a rising concern in the healthcare sector, even globally. The results highlighted that TLE, ALE, and perceived organizational support in moderating roles influence nurses' TIN.

### Limitations and recommendations

This study has made substantial research on the impact of leadership on nurses’ TIN. The study primarily targets Bahraini hospitals; future research might include the larger dimensions considering the hospitals in the Arab world. Additionally, although this study has identified a role of organizational support in a moderating role that may influence healthcare employees' intentions to leave their jobs, it still has several limitations. Future research can consider these restrictions while constructing their frameworks; thirdly, the study explores the effect of TLE and ALE on turnover while many other leadership styles go unconsidered. These factors can be emphasized in further research.

### Supplementary Information

**Additional file 1.**

## Data Availability

The datasets for this article are not publicly available due to restrictions set by the data holder. Requests to access the datasets should be directed to the authors.
