# Supplementary material for: Effect of leadership styles on turnover intention among staff nurses in private hospitals: the moderating effect of perceived organizational support
Source: BMC Health Serv Res. 2024 Feb 14;24:199. doi: 10.1186/s12913-024-10674-0 (PMC10865721; doi:10.1186/s12913-024-10674-0)
Supplement: Supplementary file 1 — Additional file 1. [file 12913_2024_10674_MOESM1_ESM.docx]

**Appendix**

**Questionnaire**

**Dear Sir / Madam**

You are invited to participate in a research study titled "Effect of Leadership Styles on Turnover Intention among Staff Nurses in Private Hospitals: The Moderating Effect of Perceived Organizational Support".  This study is being done by Surabhila Pattali, Jayendira P Sankar, Haitham Al Qahtani, Nidhi Menon, and Shabana Faizal from the College of Administrative and Financial Sciences at the University of Technology Bahrain.

The research aims to analyze the effect of leadership styles on turnover intention among staff nurses in private hospitals in Bahrain using authentic leadership style, transformational leadership style, and the moderating effect of perceived organizational support on turnover intention.

Your participation is entirely voluntary, and you can withdraw at any time.  The information provided will remain confidential and will be used only for academic research. Please note that any information you give will be treated with the utmost confidentiality and will not be used for any purpose other than this research. Your name will not be disclosed in the research output.

**Transformational Leadership Styles** [1]

| **Sl. No** | **Measured attribute** | **Statements** | **Strongly Agree**  **(5)** | **Agree (4)** | **Neutral (3)** | **Disagree (2)** | **Strongly Disagree (1)** |
| --- | --- | --- | --- | --- | --- | --- | --- |
| 1 | Idealized Influence (Behaviors) | Your manager considers the moral and ethical consequences of decisions |  |  |  |  |  |
| 2 | Inspirational Motivation | Your manager talks optimistically about the future |  |  |  |  |  |
| 3 | Intellectual Stimulation | Your manager reexamines critical assumptions to question whether they are appropriate. |  |  |  |  |  |
| 4 | Individualized | Your manager helps others to develop their strengths |  |  |  |  |  |

**Authentic Leadership Style** [2]

| **Sl. No** | **Measured attribute** | **Statements** | **Strongly Agree**  **(5)** | **Agree (4)** | **Neutral (3)** | **Disagree (2)** | **Strongly Disagree (1)** |
| --- | --- | --- | --- | --- | --- | --- | --- |
| 1 | Self -Awareness | To what degree is the leader aware of his or her strengths and limitations, how others see him or her, and how the leader impacts others? |  |  |  |  |  |
| 2 | Transparency | To what degree does the leader reinforce a level of openness with others that provides them with an opportunity to be forthcoming with their ideas, challenges and opinions? |  |  |  |  |  |
| 3 | Ethical/Moral | To what degree does the leader set a high standard for moral and ethical conduct? |  |  |  |  |  |
| 4 | Balanced Processing | To what degree does the leader solicit sufficient opinions and viewpoints prior to making important decisions? |  |  |  |  |  |

**Perceived Organizational Support** [3]

| **Sl. No** | **Statements** | **Strongly Agree**  **(5)** | **Agree (4)** | **Neutral (3)** | **Disagree (2)** | **Strongly Disagree (1)** |
| --- | --- | --- | --- | --- | --- | --- |
| 1 | The organization values my contribution to its well-being. |  |  |  |  |  |
| 2 | The organization fails to appreciate any extra effort from me. |  |  |  |  |  |
| 3 | The organization would ignore any complaint from me. |  |  |  |  |  |
| 4 | The organization really cares about my well-being. |  |  |  |  |  |
| 5 | Even if I did the best job possible, the organization would fail to notice. |  |  |  |  |  |
| 6 | The organization cares about my general satisfaction at work. |  |  |  |  |  |
| 7 | The organization shows very little concern for me. |  |  |  |  |  |
| 8 | The organization takes pride in my accomplishments at work. |  |  |  |  |  |

**Turnover Intention** [4]

| **Sl. No** | **Statements** | **Strongly Agree**  **(5)** | **Agree (4)** | **Neutral (3)** | **Disagree (2)** | **Strongly Disagree (1)** |
| --- | --- | --- | --- | --- | --- | --- |
| 1 | I intend to ask people about new job opportunities. |  |  |  |  |  |
| 2 | My current job is addressing my important personal needs. |  |  |  |  |  |
| 3 | The opportunities to achieve my most important goals at work are rarely jeopardized. |  |  |  |  |  |
| 4 | I intend to search for a position with another employer. |  |  |  |  |  |
| 5 | I occasionally think about leaving this organization. |  |  |  |  |  |
| 6 | Very often, I think about becoming an entrepreneur. |  |  |  |  |  |

1. Alban-Metcalfe RJ, Alimo-Metcalfe B (2000) The transformational leadership questionnaire (TLQ-LGV): a convergent and discriminant validation study. Leadersh Organ Dev J 21:280–296. https://doi.org/10.1108/01437730010343077

2. Cervo CS, Mónico L dos SM, Santos NR dos, et al (2016) Authentic leadership questionnaire: Invariance between samples of Brazilian and Portuguese employees. Psicol Reflex e Crit 29:1–11. https://doi.org/10.1186/s41155-016-0046-4

3. Kurtessis JN, Eisenberger R, Ford MT, et al (2017) Perceived Organizational Support: A Meta-Analytic Evaluation of Organizational Support Theory. J Manage 43:1–31. https://doi.org/10.1177/0149206315575554

4. Bothma CFC, Roodt G (2013) The validation of the turnover intention scale. SA J Hum Resour Manag 11:1–12. https://doi.org/10.4102/sajhrm.v11i1.507
